# What is Really Refractory Intracranial Hypertension in The Paediatric Group in 2025? Suggestions for ICP and CPP Guidance for Early Intervention in Malaysia

**DOI:** 10.21315/mjms-01-2025-025

**Published:** 2025-02-28

**Authors:** Shah Ozair Shaharuddin, Jafri Malin Abdullah

**Affiliations:** 1 Department of Neurosciences, School of Medical Sciences, Universiti Sains Malaysia, Health Campus, Kelantan, Malaysia; 2 Brain and Behaviour Cluster, School of Medical Sciences, Universiti Sains Malaysia, Health Campus, Kelantan, Malaysia; 3 Department of Neurosciences and Brain Behaviour Cluster, Universiti Sains Malaysia Specialist Hospital, Universiti Sains Malaysia, Health Campus, Kelantan, Malaysia

Dear Editor,

We read with great interest the article entitled “Prognostic Factors of Severe Traumatic Brain Injury Outcome in Children Aged 2–16 Years at a Major Neurosurgical Referral Centre”, which was published in the Malaysian Journal of Medical Sciences in 2009 (1).

Sixteen years since the publication of the article, multiple published guidelines have concurred that a standard intracranial pressure (ICP) of above 20 mmHg indicates a poor prognostic outcome across paediatric population (2–8). The wide age range from birth to adulthood makes the ICP cut-off point becomes physiologically precarious in the management of neonates, infants, and young children in the intensive care unit (ICU) (2, 3, 9). However, to date, no ICP threshold value has been established for each age group in the paediatric population.

We highlight that recent advanced studies have evidently shown via decay curves that paediatric patients are less tolerant of ICP increases than adult patients (7–9). Using pressure-time-dose studies, poor outcomes were extrapolated in paediatric patients with 30-minute ICPs > 15 mmHg and 150-minute ICPs >10 mmHg across all age groups (9).

As shown in Figure 1, we hypothetically postulate that a more aggressive treatment should be administered 5 minutes before the 30-minute threshold for elevated ICPs > 15 mmHg and 10 minutes before the 150-minute threshold for elevated ICP > 10 mmHg. However, continuous management of intracranial hypertension should be performed at any time to avoid prolonged elevated ICP (2, 3, 10–12).

Therefore, we strongly support the need to reduce the elevated ICP threshold for ICU paediatric patients. For the past 30 years, Malaysia has seen a significant paradigm shift in ICP management for paediatric patients with traumatic brain injury, especially towards improving the future outcome of childhood brain trauma, which is common in the country.

In addition, cerebral perfusion pressure (CPP) is a key factor that influences the outcome of traumatic brain injury. Recent studies have concluded that paediatric patients should have a CPP > 40 mmHg. Figure 1 shows the target CPP ranges for two paediatric age groups (5, 13–15).

We acknowledge that recently published studies have reported that CPPopt, ΔCPP, and PRx have predictive values for the outcome of traumatic brain injury (5). Thus, we encourage further studies to provide evidence to support their findings in paediatric populations with traumatic brain injury.

Figure 1 summarises CPP values and ICP values. Target CPPs (CPP 6 to 17 years, and CPP 0 to 5 years) (3, 4, 15). CPP Threshold is, in actuality, a single value of 40mmHg, which should be avoided as values lower than this carry a strong, independent risk factor for poor outcome (2). “Physiological” ICP for teens, children, and infants (16, 17) are shown as a guide and comparison to ICP threshold. ICP threshold constitutes a set of values of ICP above 15 mmHg for 30 minutes (dashed line A) and ICP above 10 mmHg for 150 minutes (dashed line B) are not tolerated by the paediatric age group, and the timing of intervention shall be taken within this period of time (thick black arrows) to control the ICP (9) (dashed line C).

## Figures and Tables

**Figure 1:**
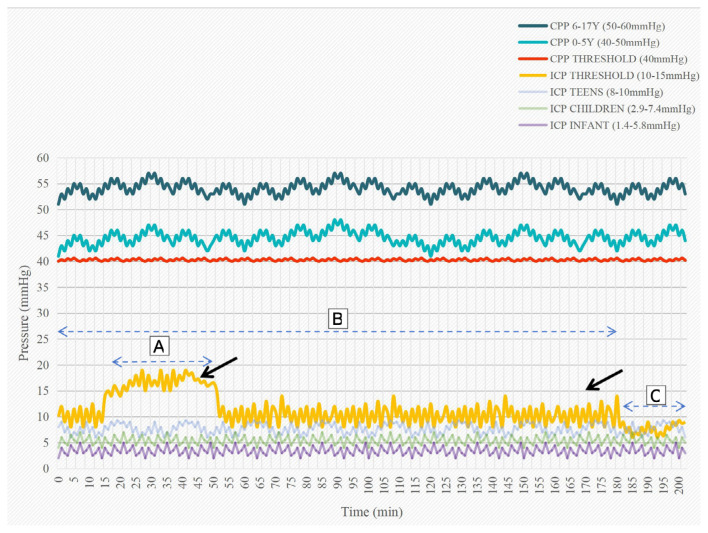
Summary of CPP values and ICP values
